# Consensus datasets of mouse miRNA-mRNA interactions from multiple online resources

**DOI:** 10.1016/j.dib.2017.07.035

**Published:** 2017-07-20

**Authors:** Zhao Bi, Bin Xue

**Affiliations:** Department of Cell Biology, Microbiology and Molecular Biology, School of Natural Sciences and Mathematics, College of Arts and Sciences, University of South Florida, 4202 East Fowler Ave. ISA2015, Tampa, FL 33620, USA

## Abstract

MiRNAs regulate gene expression by forming base pairing with mRNAs to inhibit the translation of those mRNAs. In many mammalian genomes each, about 2000 miRNAs were found to regulate roughly 60% of all the genes in that genome. Many experimental validations and computational predictions have been done on miRNA:mRNA interactions. Nonetheless, the interactions from different sources are not always consistent. In this study, we integrated multiple online resources, including mirTarBase, TarBase, miRanda, miRDB, PITA, and TargetScan, and developed eleven large-scale datasets containing miRNA:mRNA interactions that are consistent among a specific subgroup of above-mentioned online resources. In addition, a new integrated confidence score was designed to show the significance for all the miRNA:mRNA interactions.

**Specifications Table**

**Table:** 

Subject area	*Biology*
More specific subject area	*Genomics, non-coding RNA, miRNA, gene expression*
Type of data	*Excel file*
How data was acquired	*Databases*
Data format	*Raw, filtered, analyzed*
Experimental factors	*Experimentally validated data were incorporated*
Experimental features	*Consistent data were selected from multiple sources*
Data source location	*Online resources*
Data accessibility	*Within this article*

**Value of the data**

• The eleven datasets are the largest consistent datasets of miRNA:mRNA interactions containing both true and false samples in mouse genome.
• These datasets can be further refined as training and test datasets in the development of new miRNA target predictors, and therefore are critical for bioinformatics studies.
• The samples in these datasets are genome-wide and are consistent among multiple sources. Therefore, these datasets can be used directly to extract high-confidence miRNA:mRNA interactions.

## 1 Data

MiRNAs are short non-coding RNA molecules with about 22 nucleotides. Although being very short compared to mRNAs, which normally have hundreds to thousands of nucleotides, miRNAs may interact with the 3′-UTRs of mRNAs to inhibit the translation of those mRNAs. MiRNAs are abundant in natural, there are more than 2000 miRNAs in each mammalian genome, regulating about 60% of all the genes in that genome. Clearly, miRNAs are a critical family of gene expression regulators. However, a thorough understanding on the function and mechanism of miRNAs is still elusive. To facilitate the studies of miRNA molecular biology, curated datasets of miRNA:mRNA interactions will be very helpful. The eleven datasets developed in this project are the largest consistent datasets containing both true and false miRNA:mRNA interactions in mouse genome. An integrated confidence score has also been designed to show the significance of these interactions.

## 2 Experimental design, materials and methods

The consistent datasets were developed using the following online resources: miRNA:mRNA interaction data of mouse genome in mirTarBase Release 6.0 [1] and TarBase v7.0 [2], pre-assembled datasets for predicted miRNA:mRNA interactions in mouse genome downloaded from the web servers of miRanda 2010 release [3], miRDB v5.0 [4], PITA v6 [5], and TargetScan 7.0 [6].

MiRTarBase and TarBase are the most popular databases of experimentally validated miRNA:mRNA interactions. These two databases contain comprehensive data of miRNA:mRNA interactions from both dependent and independent sources, and can be downloaded without any restrictions. For these two reasons, both of the databases were selected as the source of experimentally validated miRNA:mRNA interactions. The experimental methods referenced in mirTarbase and TarBase include about 30 different types, such as: reporter assay, western blot, Cross-Linking Immunoprecipitation (CLIP), etc.

Predicted miRNA:mRNA interactions were downloaded from the web servers of miRanda, miRDB, PITA, and TargetScan. The reasons for choosing these four computational predictors are: (1) These predictors are well-designed miRNA target predictors using synthesized techniques. The original version of miRanda predicts miRNA targets based on sequence match, free energy calculated from Vienna RNA package, and evolutionary conservation verified from sequence alignment [3]. Nonetheless, the newest version of miRanda has integrated an mirSVR score into the output prediction [7]. The mirSVR score is the output of “a support vector regression approach to model the degree of microRNA regulation given a set of numerical features representing the microRNA binding site and additional contextual information” [7]. The mirSVR score is actually a measure of the changes of logarithm-based expressional levels of down-regulated mRNAs upon miRNA transfection [7]. The results in miRDB were predicted using mirTarget2, a support vector machine based predictor [8]. The final score of miRDB ranges from 0 to 100 and shows the relative significance of predicted target genes [9]. PITA predicts targets based on sequence match between mRNA and miRNA calculated from RNAduplex [10], and secondary structure of mRNA predicted using RNAFold [10]. The PITA prediction score is an energetic score showing the free-energy change upon the miRNA:mRNA binding [5]. The predictive results of PITA can be further filtered to keep conserved sequences by using phastCons, which is built on hidden Markov model [5], [11]. In this study, conserved PITA predictions were used and analyzed. TargetScan was designed to search for the conserved sequence complementarity between the seed region of miRNA and the 3’-UTR of mRNA [6]. The Pct score (Probability of Conserved Targeting) [12] from TargetScan was used to evaluate the significance of the prediction. The default threshold values of positive prediction are <-1.0, >80, <-10, and >0.36, for miRanda, miRDB, PITA, and TargetScan, respectively. And vice versa for negative predictions. (2) These predictors were developed or upgraded in recent years, and are well-maintained; (3) These predictors have been broadly used in this field; (4) The web servers of these predictors provide pre-assembled dataset of predicted miRNA:mRNA interactions of mouse genome.

For each pair of miRNA and mRNA molecules, the above-mentioned predictors may make either a valid prediction or an invalid prediction. The difference between valid prediction and invalid prediction is that there is no meaningful output in an invalid prediction, while the valid prediction is always accompanied by a determinative conclusion of the prediction. For this reason, all the predicted miRNA:mRNA interactions download from miRanda, miRDB, PITA, and TargetScan web servers were examined to keep only valid predictions. Since each of these valid predictions could be four-predictor-overlapped or 3-predictor-overlapped or two-predictor-overlapped, this valid prediction was then saved in a corresponding dataset based on which predictors have valid predictions. There are in total C(4,4) + C(4,3) + C(4,2) = 1 + 4 + 6 = 11 datasets as shown in Table 1. It should be noted that the number of miRNA:mRNA interaction pairs that can be validly predicted by only one predictor is very limited and therefore these interactions are not included in this study. Afterwards, for each of the afore-mentioned eleven datasets, all the miNRA:mRNA pairs in that dataset were compared with the miRNA:mRNA pairs in the mirTarBase and TarBase databases. If the miRNA:mRNA pair can be found in mirTarBase or Tarbase, the miRNA;mRNA pair is assigned as a true sample. If the pair can’t be found in mirTarBase and TarBase, the prediction scores of this miRNA:mRNA pair given by corresponding predictors were compared to their default threshold values. If all the predictions are negative, this miRNA:mRNA pair is assigned as false sample. Otherwise, the miRNA:mRNA pair is excluded from further analysis. The eleven datasets, and corresponding numbers of true and false samples of these eleven datasets are shown in Table 1.

**Table 1:** Summary of the eleven datasets.

Dataset ID	Associated predictors	No. of true samples	No. of false samples
D4	miRanda, miRDB, PITA, TargetScan	1739	9870
D3-1	miRanda, miRDB, PITA	1423	11,062
D3-2	miRanda, miRDB, TargetScan	195	2667
D3-3	miRanda, PITA, TargetScan	2997	43,082
D3-4	miRDB, PITA, TargetScan	1990	24,088
D2-1	miRanda, miRDB	187	2722
D2-2	miRanda, PITA	3817	177,467
D2-3	miRanda, TargetScan	423	21,858
D2-4	miRDB, PITA	1713	14,264
D2-5	miRDB, TargetScan	1638	189,900
D2-6	PITA, TargetScan	6211	1340
TOTAL		22,333	498,320

**Fig. 1:**
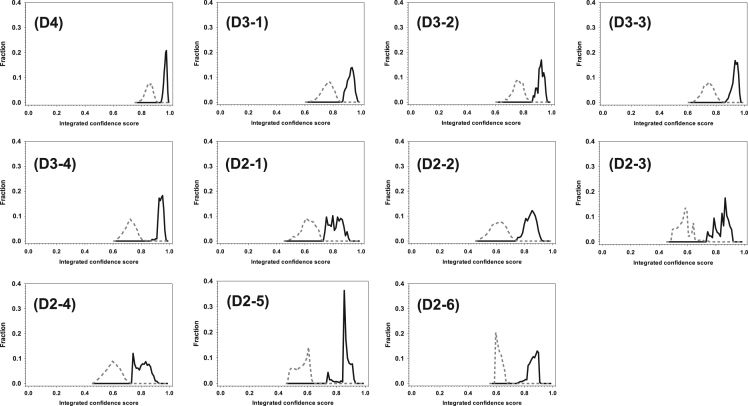
Distribution of both true and false samples as a function of integrated confidence score in eleven datasets. X-axes show the integrated confidence score. Y-axes present the fractions of true samples (solid lines) and false samples (dashed lines) in each interval of integrated confidence score.

All the miRNA:mRNA pairs in each of the eleven datasets have a specific number of predictive scores given by specific predictors. Therefore, for a miRNA:mRNA pair in a specific dataset, a confidence score was calculated by the following procedure: Assume the i-th sample in this dataset has S predictive scores $v_{i}^{s}$, s=1,…, S. S is the total number of predictors associated with this dataset. By using the predictive scores from the s-th predictor for all the samples in this dataset, the z-scores for all the samples were calculated. Then the p-values were calculated using scipy package of python. Afterwards, the i-th sample in the dataset has S p-values, $p_{i}^{s}$, s=1,…,S. Each of these p-values was then transformed to $d_{i}^{s}=1/(1+e^{-p_{i}^{s}})$ . Finally, if the sample is a true sample, the integrated confidence score is calculated using $C_{i}=1-\prod_{1}^{S}(1-d_{i}^{s})$ ; if the sample is a false sample, the integrated confidence score is determined by $C_{i}=1-\prod_{1}^{S}d_{i}^{s}$ . The distributions of integrated confidence scores for both true and false samples in the eleven datasets were presented in Fig. 1. Clearly, different datasets have different ranges of independent variables and dependent variables. For the purpose of showing the difference, we didn’t pursue the renormalization of the distribution. A subset of these datasets was refined and applied in one of our recent studies on decision-tree based new algorithm of miRNA target prediction [13].

## References

[1] Chou C.H., Chang N.W., Shrestha S., Hsu S.D., Lin Y.L., Lee W.H., Yang C.D., Hong H.C., Wei T.Y., Tu S.J. (2016). miRTarBase 2016: updates to the experimentally validated miRNA-target interactions database. Nucleic acids Res..

[2] Vlachos I.S., Paraskevopoulou M.D., Karagkouni D., Georgakilas G., Vergoulis T., Kanellos I., Anastasopoulos I.L., Maniou S., Karathanou K., Kalfakakou D. (2015). DIANA-TarBase v7.0: indexing more than half a million experimentally supported miRNA:mrna interactions. Nucleic acids Res..

[3] Enright A.J., John B., Gaul U., Tuschl T., Sander C., Marks D.S. (2003). MicroRNA targets in Drosophila. Genome Biol..

[4] Wong N., Wang X. (2015). miRDB: an online resource for microRNA target prediction and functional annotations. Nucleic acids Res..

[5] Kertesz M., Iovino N., Unnerstall U., Gaul U., Segal E. (2007). The role of site accessibility in microRNA target recognition. Nat. Genet..

[6] Lewis B.P., Burge C.B., Bartel D.P. (2005). Conserved seed pairing, often flanked by adenosines, indicates that thousands of human genes are microRNA targets. Cell.

[7] Betel D., Koppal A., Agius P., Sander C., Leslie C. (2010). Comprehensive modeling of microRNA targets predicts functional non-conserved and non-canonical sites. Genome Biol..

[8] Wang X., El Naqa I.M. (2008). Prediction of both conserved and nonconserved microRNA targets in animals. Bioinformatics.

[9] Wang X. (2016). Improving microRNA target prediction by modeling with unambiguously identified microRNA-target pairs from CLIP-ligation studies. Bioinformatics.

[10] Lorenz R., Bernhart S.H., Honer Zu Siederdissen C., Tafer H., Flamm C., Stadler P.F., Hofacker I.L. (2011). ViennaRNA Package 2.0. Algorithms Mol. Biol.: AMB.

[11] Siepel A., Haussler D., Nielsen R. (2005). Phylogenetic hidden Markov models. Statistical Methods in Molecular Evolution.

[12] Friedman R.C., Farh K.K., Burge C.B., Bartel D.P. (2009). Most mammalian mRNAs are conserved targets of microRNAs. Genome Res..

[13] Zhao B., Xue B. (2017). Improving prediction accuracy using decision-tree-based meta-strategy and multi-threshold sequential-voting exemplified by miRNA target prediction. Genomics.

